# Geographic variation in sexual communication in the cotton bollworm, *Helicoverpa armigera*

**DOI:** 10.1002/ps.5893

**Published:** 2020-06-10

**Authors:** Ke Gao, Luis M Torres‐Vila, Myron P Zalucki, Yiping Li, Frans Griepink, David G Heckel, Astrid T Groot

**Affiliations:** ^1^ Institute for Biodiversity and Ecosystem Dynamics University of Amsterdam Amsterdam The Netherlands; ^2^ Servicio de Sanidad Vegetal Consejería de Medio Ambiente y Rural PAyT Badajoz Spain; ^3^ School of Biological Science The University of Queensland Brisbane Australia; ^4^ Key Laboratory of Plant Protection Resources and Pest Management, Ministry of Education Northwest A&F University Yangling China; ^5^ Pherobank BV Wijk bij Duurstede The Netherlands; ^6^ Max Planck Institute for Chemical Ecology Department of Entomology Jena Germany

**Keywords:** sexual behavior, sex pheromone, communication interference, cotton bollworm

## Abstract

**BACKGROUND:**

Geographic variation in male response to sex pheromone lures has been studied in the field in a number of moth species. However, only a few studies have investigated geographic variation in female calling and sex pheromone under field conditions. For an effective field implementation of sex pheromone lures, it is essential to know the local sex pheromone blend and local timing of sexual communication. We investigated the level and extent of geographic variation in the sexual communication of the important agricultural pest *Helicoverpa armigera* (Lepidoptera, Noctuidae) in three continents.

**RESULTS:**

We found there is no genetic variation in the calling behavior of *H. armigera*. In the female sex pheromone, we found more between‐population variation than within‐population variation. In male response experiments, we found geographic variation as well. Strikingly, when adding the antagonistic compound Z11‐16:OAc to the pheromone blend of *H. armigera*, significantly fewer males were caught in Australia and China, but not in Spain. This variation is likely not only due to local environmental conditions, such as photoperiod and temperature, but also to the presence of other closely related species with which communication interference may occur.

**Conclusion:**

Finding geographic variation in both the female sexual signal and the male response in this pest calls for region‐specific pheromone lures. Our study shows that the analysis of geographic variation in moth female sex pheromones as well as male responses is important for effectively monitoring pest species that occur around the globe. © 2020 The Authors. *Pest Management Science* published by John Wiley & Sons Ltd on behalf of Society of Chemical Industry.

## Graphical abstract

In this paper we found geographic variation in the female sex pheromone and the male response of the cotton bollworm, *Helicoverpa armigera*.

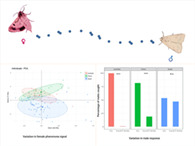

## 1 INTRODUCTION

To reduce the use of chemical insecticides against pests in agriculture, several environmentally friendly approaches have been developed in recent years.^1^, ^2^, ^3^ One effective method is the behavioral manipulation of pest species.^4^ For example, manipulation of sexual communication of insects has been widely used in integrated pest management, from monitoring to controlling pest populations through mate disruption.^5^, ^6^, ^7^ However, the effectiveness of behavioral manipulation methods can be greatly increased by taking into account behavioral and ecological variation of the pests, especially for pests with wide geographical distributions.^8^

Geographic variation in sexual communication of insects is a common phenomenon.^9^, ^10^, ^11^, ^12^, ^13^ For an effective field implementation, it is essential to know the local sex pheromone blend and local timings of sexual communication. Variation in timing of sexual behaviors in moths, such as female calling and pheromone release, has been extensively studied under laboratory conditions, showing that these sexual behaviors are affected by abiotic factors, such as photoperiod,^14^, ^15^, ^16^ temperature,^17^ relative humidity^18^, ^19^ and wind speed.^20^ Sexual behaviors in moths may also be affected by biotic and physiological factors, such as age,^21^, ^22^, ^23^ host plants,^24^, ^25^ larval diet,^26^ pupal period^27^ and insecticides.^28^, ^29^ In addition, variation in moth sexual communication may be caused through communication interference between closely related species in areas of sympatry.^13^, ^30^, ^31^ Although moth sex pheromone signals are species‐specific, closely related species often share common sex pheromone components and some components in the pheromone blend may play roles as antagonists to avoid heterospecific attraction.^10^, ^13^, ^32^

Geographic variation in male response to pheromone lures has been studied in the field in a number of species.^10^, ^33^, ^34^, ^35^ However, only a few studies have investigated geographic variation in female calling and sex pheromone release under natural conditions in the field, i.e. under local prevailing temperatures, photoperiodic conditions and the presence of other related species.^36^, ^37^, ^38^ Consequently, our understanding of how abiotic and biotic factors under natural conditions affect sexual communication in moths is still scarce, particularly under rapid environmental changes.^39^

The cotton bollworm, *Helicoverpa armigera* (Hübner), is an important multivoltine pest, occurring throughout Africa, Europe, Asia and Oceania,^40^ and has a long history of worldwide insecticide resistance to several chemicals.^41^ Recently, *H. armigera* has been found as an invasive species and causes significant economic losses in South and Central America^42^, ^43^, ^44^ and threatens to spread further.^45^ The larvae are highly polyphagous and feed on a variety of host plants, including economically important crops, such as cotton, corn, soybean and tomato.^46^ In addition, *H. armigera* has the ability to undergo facultative diapause and seasonal migration with long‐distance dispersal.^47^ Among *H. armigera* populations, geographic variation in host plant preference have been found,^48^, ^49^ but as far as we know geographic variation in sexual communication has not been documented yet.

Application of sex pheromone has become increasingly important for integrated pest management of *H. armigera* throughout the world.^6^, ^50^ In this study, we investigated the level and extent of geographic variation in the sexual communication of the moth *H. armigera*, combining field experiments under natural conditions and laboratory experiments. To determine geographic variation in the timing of female calling and sex pheromone production, and male attraction to different synthetic pheromone blends we investigated *H. armigera* populations in Spain, China and Australia. To determine the consistency of circadian rhythms of female sexual activities, we also assessed variation in the timing of female calling in the laboratory.

## 2 MATERIALS AND METHODS

### 2.1 Field locations

**Figure 1:**
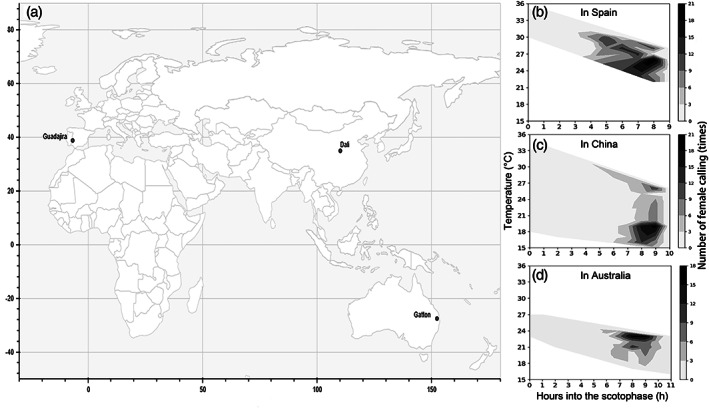
Field experiments conducted in different geographic regions. (a) Field locations. (b)–(d) Correlations between timing of female calling and temperature in the three different regions. Shaded areas showed the measured fluctuations of temperature during the observation nights.

Field experiments were conducted between 2016 and 2017 during *H. armigera* flight seasons in three continents: Guadajira, Badajoz, Spain, which is a major processing tomato growing area; Dali County, Shaanxi, China, which is a cotton growing area; and Gatton, Brisbane, Australia, which is a mixed horticultural cropping area (Fig. 1(a) and Table 1).

**Table 1:** Geographic locations of *H. armigera* populations studied in the field

Country	Location	Coordinates	Main crop	Date	Photoperiod
Spain	Guadajira, Badajoz	38° 51′ 08.8″ N, 6° 40′ 48.9″ W	Tomato	June–August 2016	15 L:9D
China	Dali county, Shaanxi	34° 45′ 01.9″ N, 110° 09′ 56.1″ E	Cotton	July–September 2017	14 L:10D
Australia	Gatton, Brisbane	27° 32′ 11.6″ S, 152° 20′ 16.3″ E	Lucerne	January–March 2017	13 L:11D

### 2.2 Effects of larval diet (host plants) on sexual communication

To compare within‐population variation to between‐population (i.e. geographic) variation, at each field site we compared female calling and sex pheromone amount and composition from females that were reared as larvae on three different host plants per site in semi‐outdoor conditions. At each field site, we collected 50–90 gravid *H. armigera* females with an entomological net. These field‐collected females were placed singly in plastic beakers (473 mL; Solo, Lake Forest, IL, USA) provided with 10% sugar water and covered with a gauze cloth, on which the females oviposited eggs. After the eggs hatched, the larvae were placed individually into transparent plastic cups (37 mL; Solo) and supplied with fruits of local host plants, i.e. tomato, corn and pepper in Spain, and tomato, corn and cotton in China and Australia. The host plants were replaced every day until pupation. Pupae were checked daily for emerging adults, and these were sexed and placed separately into transparent cups (37 mL) containing wool cotton soaked with 10% sugar water. Newly emerged virgin females were used in the calling observations and pheromone extractions at each field site.

### 2.3 Female calling behavior

To evaluate variation in the time of female calling behavior at each field site, the newly emerged virgin females were placed singly in clear transparent plastic beakers (473 mL) covered with a gauze cloth. We kept track on the host plants on which the females had been reared. The night of emergence was defined as age 0. The observations started with 1‐day‐old females and were repeated on successive nights. The age at which females initiated calling, as well as the duration of calling behavior, were observed every 30 min with a red light throughout the night, i.e. from sunset to sunrise. Female calling behavior was noted when the ovipositor with pheromone gland was clearly extruded from the abdomen. During the observational nights, fluctuations in temperature and relative humidity were recorded by a hygrothermograph (TFA Dostmann, Wertheim, Germany) at 1 h intervals.

To assess whether variation in female calling behavior was due to geographic differences, calling behavior was also determined in the laboratory. For these experiments, eggs and larvae of *H. armigera* in the fields were collected from the same three field sites in Spain, China and Australia in 2018, and shipped to the laboratory at the University of Amsterdam, where they were reared individually on artificial pinto bean diet in climate chambers [60% relative humidity (RH); 25 °C; 14 h light:10 h dark with lights off at 11 am]. Upon hatching, newly emerged adults were sexed and placed separately into transparent cups (37 mL) containing cotton wool soaked with 10% sugar water. In this study, 3‐ to 6‐day old virgin females from each population were observed in the climate chamber in which the larvae were reared. These observations were conducted every 30 min with a red light throughout scotophose (10 h from 11 am to 9 pm) and repeated for two consecutive nights.

### 2.4 Female pheromone analysis

To compare within‐population variation to between‐population variation in the sex pheromone quantity and composition, female pheromone glands were extracted individually from the different groups of females that were reared on the different diets, after the females were used for calling observations in each region. Glands were extracted at 2‐hour intervals throughout the night after all observation of calling. As the night in Spain lasted only 9 h, glands were extracted at four timepoints (1, 3, 5 and 7 h into the night). In China and Australia, the night lasted 10 and 11 h, respectively, so that in these regions glands were extracted at five timepoints (1, 3, 5, 7 and 9 h into the night). At each time point, glands were dissected with fine scissors and forceps, and deposited individually in conical vials. The conical vials, containing 200 ng pentadecane within 50 μL hexane as internal standard, had been prepared in advance at the laboratory in GC vials with Alu Crimp caps (11 mm) and spring inserts, which minimizes evaporation and allowed the extracts to be transported. After 30–40 min, the glands were removed from the solution with forceps, and the extracts were sealed and kept at −20 °C until shipping.

All pheromone samples were analyzed at the University of Amsterdam, in a HP7890 Gas Chromatograph (GC) with a 7683 automatic injector, as detailed in Groot *et al*.^9^ and summarized here. The hexane extracts were reduced to 2 μL under a gentle stream of N_2_, after which each sample with 1 μL octane was injected into the GC. The sex pheromone peaks were identified and integrated based on their retention times, which were compared to a synthetic pheromone blend of *H. armigera*, which was injected before and after each round of 30 injections. The amount of each pheromone component was calculated relative to the 200 ng of internal standard.

### 2.5 Male response experiments

To test geographic variation in male attraction to pheromone lures in different regions, two field trapping experiments, each with two to four replicates were conducted at each of the three sites in a complete randomized block design (Table 2). In experiment 1, the attraction of five synthetic pheromone blends was compared, which were prepared at Pherobank BV (Wijk bij Duurstede, the Netherlands). Each pheromone lure consisted of a red rubber septum that was loaded with 100 μL of hexane containing 5 mg of the major component Z11‐16:Ald (100%), while the other compounds were loaded in amounts relative to Z11‐16:Ald (see Table 2). In experiment 2, the effect of the antagonist pheromone compound Z11‐16:OAc on *H. armigera* male attraction was tested. We focused on this antagonistic compound because it is part of the sex pheromone blend of the closely related species *H. assulta* that occurs sympatrically with *H. armigera* in China.^51^ For experiment 2, we prepared two synthetic pheromone lures in the laboratory. Each pheromone lure consisted of a red rubber septum that was loaded with 100 μL of hexane containing 300 μg of Z11‐16:Ald (100%) and other compounds relative to 300 μg of Z11‐16:Ald, so that one treatment consisted of 100% Z11‐16:Ald and 5% Z9‐16:Ald (which are the two critical sex pheromone components of *H. armigera* and to which we refer as the H.a blend), and another treatment consisted of 100% Z11‐16:Ald and 5% Z9‐16:Ald + 10% Z11‐16:OAc (H.a + Z11‐16:OAc blend) (Table 2).

**Table 2:** Compositions of pheromone lures used in the fields

Experiment	Lures	Z11‐16:Ald	Z9‐16:Ald	Z7‐16:Ald	Z9‐14:Ald	Z11‐16:OAc
**1**	Blend1	100				
	Blend2	100	2.5	0.6		
	Blend3	100	1.4		0.3	
	Blend4	100	4			
	Blend5	100	6			
**2**	H.a	100	5			
	H.a + Z11‐16:OAc	100	5			10

In experiment 1, compound concentrations were as follows: 100% = 5 mg, 2.5% = 0.125 mg, 1.4% = 0.07 mg, 4% = 0.2 mg, 6% = 0.3 mg, 0.6% = 0.03 mg, 0.3% = 0.015 mg.

In experiment 2, compound concentrations were as follows: 100% = 300 μg, 5% = 15 μg, 10% = 30 μg.

All lures were hung in bucket traps (Pherobank BV) attached to a wooden pole and positioned at a height of approximately 1.5 m above the ground, and distributed at least 30 m apart in the field. The males caught in the traps were collected and counted every day. All the lure experiments were conducted in the field in each region (Table 1). For experiment 1, at all field sites two rubber septum lures per treatment at the same time were used and the treatments were rotated daily over five nights (ten replicates per treatment) to minimize possible position and odorant effects. For experiment 2, in Spain, four rubber septum lures per treatment at the same time were used and the treatments were rotated daily over two nights (eight replicates per treatment). In Australia, two rubber septum lures per treatment at the same time were used and rotated daily over five nights (ten replicates per treatment). In China, three rubber septum lures per treatment at the same time were used and rotated daily over three nights (nine replicates per treatment).

### 2.6 Data analysis

All statistical analyses were performed in R software, version 3.4.1 (R Core Team, 2018). The effect of larval diets (host plants) on the calling behavior of females within each geographic region was tested using a generalized linear model (GLM) with a binomial distribution, where the percentage of calling was the response variable, time and larval diet were the independent variables. Difference in the age of female initial calling among the three populations was compared using a Kruskall–Wallis test, followed by Dunnʼs test for multiple comparisons. Under laboratory conditions, the calling behaviors of females among the three populations were compared using a GLM with a binomial distribution, where the percentage of calling was the response variable, and time and population were the independent variables. To compare the pheromone signal between females, we conducted the following analyses. Within each region, the effect of larval diets (host plants) on the total amount of pheromone was tested using a GLM with a negative binomial distribution. To assess geographic variation in the relative amounts of the compounds in the pheromone blend among the three populations, female pheromone data from the main calling time (i.e. the last two time points in each region) were log(*x* + 1)‐transformed to stabilize the variance and then compared using a GLM with a negative binomial distribution, after which a MANOVA analysis was performed. Each compound among the three populations was compared with ANOVA, followed by Tukey–Kramer HSD test at the 5% probability level for multiple comparisons. To compare male responses between the treatments at each field site, the number of males caught in each field site were analyzed using a generalized linear model with a Poisson distribution, followed by a Tukey–Kramer HSD test at the 5% probability level for multiple comparisons in experiment 1. The difference of males caught in experiment 2 was compared by nonparametric Wilcoxon rank‐sum test.

## 3 RESULTS

### 3.1 Variation in the timing of female calling

**Figure 2:**
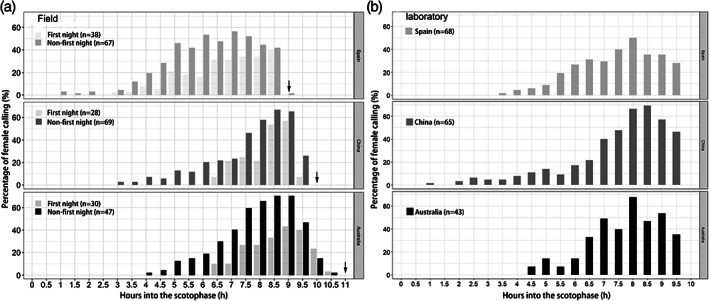
Female calling patterns (a) at the three field locations and (b) in the laboratory. The black arrows in (a) indicate the end of scotophase in Spain, China and Australia. *n*, number of females exhibiting calling behavior.

Within each population, approximately 75% of females observed exhibited calling behavior (total number of females observed: in Spain, *n* = 85; in China, *n* = 67; in Australia, *n* = 53). Larval diets (host plants) did not affect adult female calling behavior in Spain (*P* = 0.699), China (*P* = 0.921) or Australia (*P* = 0.837). Among the three populations, the age of female initial calling varied: in Spain, females started calling at significantly younger age (*n* = 38, 1.8 ± 0.1 days) compared to females in China (*n* = 28, 2.7 ± 0.2 days) (*P* = 0.005) and Australia (*n* = 30, 3.5 ± 0.3 days) (*P* < 0.001). Within all three populations, the calling patterns were significantly different between the first calling night and later calling nights (Spain: *χ* ^*2*^ = 8.41, df = 1, *P* = 0.004; China: *χ* ^*2*^ = 5.96, df = 1, *P* = 0.015; Australia: *χ* ^*2*^ = 6.71, df = 1, *P* = 0.01) (Fig. 2(a)).

In all three populations, female calling was very low in the first half of the night and then increased sharply in the second half of the night (Fig. 2(a)). A similar pattern was shown in all three regions, even though there were differences in night lengths and temperatures in the three geographic regions, i.e. *H. armigera* females showed similar temporal patterns of calling behavior, namely in the last part of the night (Figs. 1(b) and 2(a)). When we recorded the female calling behavior in all three sites, temperatures generally decreased throughout the nights. Changes in temperature during the nights were smaller in Spain (22–35 °C) and in Australia (16–27 °C), with female calling during the optimum temperature at 24–26 °C in Spain and at 20–23 °C in Australia. However, in China, even when the temperature during the nights fluctuated a bit more, namely between 15–34 °C, the females still exhibited calling behavior at lower temperature (i.e. lower temperature coincided with rainfall during the nights) (Fig. 1(b)). Under laboratory conditions, the percentage of females calling were similar among the three populations (*P* = 0.884) (Fig. 2(b)).

### 3.2 Variation in female pheromone amount and composition

**Figure 3:**
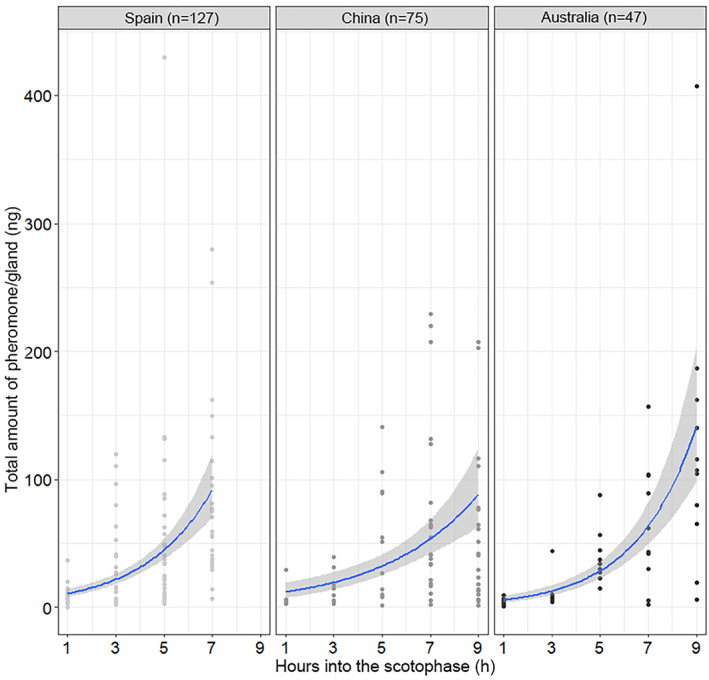
Total pheromone amounts in females (ng) when glands were extracted at different time points into the scotophase in the three field locations. The gray areas represent 95% confidence intervals. *n*, total number of females extracted.

**Figure 4:**
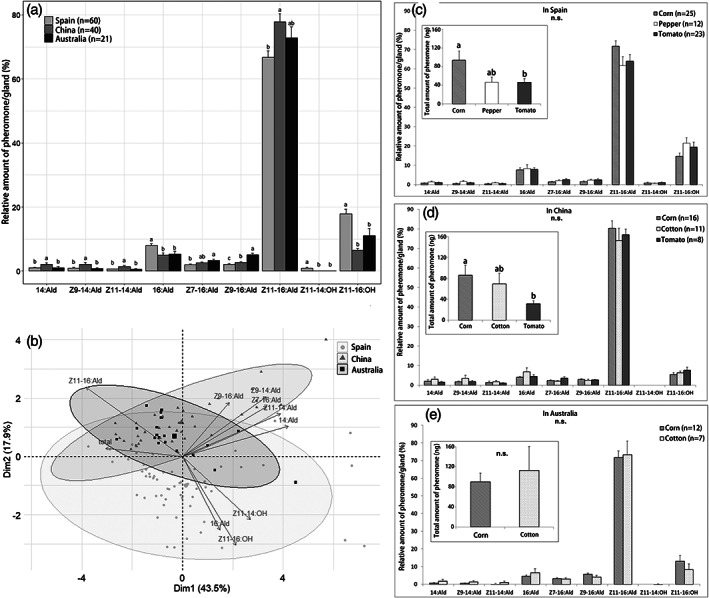
Pheromone composition of *H. armigera* females in Spain, China and Australia. (a) Relative amount (%) of female pheromone compounds. (b) Principal component analysis (PCA) of female pheromone blends. PCA plot shows the first two principal components and the arrows represent each pheromone component. (c)–(e) Quantity (in ng, inserts) and quality (in %, main panels) of pheromone blends from females reared on different larval diets (host plants) in (c) Spain, (d) China and (e) Australia. Significant differences are indicated by different letters (*P* < 0.05). n.s., not significant; *n*, total number of females extracted.

Within each population, larval diets did not affect the pattern of female sex pheromone titers, as this increased in a similar pattern throughout the night (Fig. 3). Larval diets also did not affect pheromone amounts in Australia (*χ* ^*2*^ = 0.44, df = 1, *P* = 0.51) (Fig. 4(e)). However, in Spain and China, females that were reared on corn contained significantly more pheromone than females reared on tomato (Spain: *P* = 0.015; China: *P* = 0.042) (Figs. 4(c),(d)). The pheromone composition (i.e. relative amounts) was unaffected by larval diets in all three regions: (Spain: df = 2, *P* = 0.091; China: df = 2, *P* = 0.37; Australia: df = 1, *P* = 0.051) (Figs 4(c)–(e)).

Among the populations, the pheromone compositions were significantly different (df = 2, *P* < 0.001) (Figs. 4(a),(b)). Specifically, females from China contained significantly more of the major sex pheromone component Z11‐16:Ald, and the minor compounds 14:Ald, Z9‐14:Ald and Z11‐14:Ald, than females from Spain. In contrast, females from Spain contained significantly more 16:Ald and Z11‐16:OH, while females from Australia contained significantly more Z7‐16:Ald and the critical secondary sex pheromone component Z9‐16:Ald. Interestingly, Z11‐14:OH was present in Spain, but not in China and Australia.

### 3.3 Variation in male response

**Figure 5:**
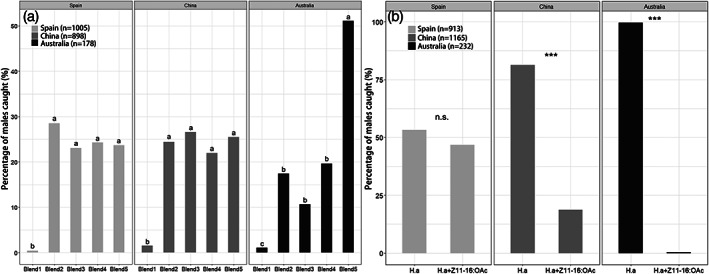
Field trapping experiments in the three field locations, showing the percentage of *H. armigera* males caught. (a) Attraction of *H. armigera* males to five blends, as specified in Table 2. (b) Attraction of *H. armigera* (H.a) males to H.a pheromone blends with or without 10% Z11‐16:OAc, as specified in Table 2. Significant differences are indicated by different letters (*P* < 0.05) or asterisks (***P < 0.001). n.s., not significant; *n*, total number of males caught.

In experiment 1, *H. armigera* males were caught in all tested traps, although traps with lures containing only the major component Z11‐16:Ald (blend 1) caught significantly fewer males in all fields compared to blends 2–5. In Spain and China, males were equally attracted to traps with blend 2–5, to which Z9‐16:Ald, Z7‐16:Ald or Z9‐14:Ald was added. However, in Australia about twice as many males were caught in blend 5 containing 6% Z9‐16:Ald than in blends 2–4 (Fig. 5(a)).

In experiment 2, the addition of 10% Z11‐16:OAc significantly reduced the number of males captured in Australia (*P* < 0.0001) and China (*P* < 0.0001), but not in Spain, where equal numbers of males were caught in traps with or without this acetate ester (*P* = 0.599) (Fig. 5(b)).

## 4 DISCUSSION

Although *H. armigera* is widely distributed across a large latitudinal gradient and thus experiences different photoperiodic conditions, we found that overall female calling behavior was similar among the three geographic regions. However, we did find that *H. armigera* females in Spain initiated calling at a significantly younger age than in Australia and China. This is likely due to the fact that in Spain nights were shorter and the temperature at night was higher compared to China and Australia (Fig. 1(b)). Previous studies also found that photoperiod and temperature affect the age that females initiate calling.^14^, ^52^ This variation hints at physiological differences in sexual maturation, which could be an adaptation to changes in different photoperiodic and temperature conditions, especially for migratory species.^22^, ^52^, ^53^

Previous studies have suggested that there could be genetic variation in the calling behavior of *H. armigera*.^38^, ^54^ However, as we did not find differences in the timing of calling among the *H. armigera* populations in Spain, China and Australia under laboratory conditions, genetic variation in calling behavior is unlikely, at least for these populations.

In all three geographic sites, the total amount of pheromone increased throughout the night, which reflects the calling behavior patterns. Such a synchronization between female calling and pheromone release has been previously shown in many other moth species from several families, i.e. *Helicoverpa assulta* (Noctuidae), *Phthorimaea operculella* (Gelechiidae), *Agrotis ipsilon* (Noctuidae) and *Choristoneura rosaceana* (Tortricidae).^16^, ^55^, ^56^, ^57^

Within each geographic region, we found that larval diet did not affect the composition of the pheromone blends, but it did affect the total amount of pheromone. Larval nutrition can directly influence pupal weight or adult body size, which has been found to be positively correlated with pheromone titer.^24^, ^58^ Since females that were reared on corn produced more pheromone than females reared on tomato both in Spain and China (Figs. 4(c),(d)), possibly females that were reared on corn acquired more nutrition than larvae fed on tomato. Alternatively, geographic variation in host plant suitability may induce larval stress, which may indirectly affect adult pheromone amounts.^59^ Although *H. armigera* can utilize a large range of host plants, host preference and suitability varies in different geographic regions. For example, *H. armigera* in Australia prefers tobacco and corn,^60^ while tomato is the main host plant in Spain.^41^ Interestingly, tomato seems an unsuitable host for *H. armigera* in China.^61^

Among the three geographic regions, we found some differences in the relative amounts of the pheromone compounds, especially between the populations from Spain and China. Specifically, we found a significantly lower amount of the major pheromone component Z11‐16:Ald, but higher amount of the minor compounds 16:Ald, Z9‐16:Ald and Z11‐16:OH in Spain than in China. In addition, we detected a very low amount of Z11‐14:OH in females from Spain, but not in China and Australia. Konyukhov *et al*.^62^ first reported the presence of Z11‐14:OH in *H. armigera* female pheromone blends. However, further study will be needed to confirm the biological function and relevance of Z11‐14:OH in *H. armigera* female pheromone glands.

One possible explanation for the geographic variation in the relative amounts of some pheromone compounds could be potentially interference between closely related species.^13^, ^63^ In China *H. armigera* co‐occurs with *H. assulta*, and in Australia with *H. punctigera*. Selection may be exerted to reduce cross‐attraction and hybridization in these areas of sympatry, so that females vary the relative amounts of pheromone compounds.^10^, ^64^ For example, tuning the major compounds Z11‐16:Ald or Z9‐16:Ald or adding other components could increase the attraction of intraspecific males in different regions.^65^, ^66^

We also found geographic variation in male response to different pheromone lures. In general, the two pheromone components for *H. armigera* are Z11‐16:Ald and Z9‐16:Ald, and the combination of these two components is recommended as the standard blend for attracting the species.^67^, ^68^ Zhang *et al*.^69^ found that the addition of Z9‐14:Ald into the standard blend attracted more males, while the addition of Z7‐16:Ald did not increase attraction. However, the results of our field tests showed that neither Z9‐14:Ald nor Z7‐16:Ald increased the attraction of males in any of the three sites. Interestingly, the addition of 6% of Z9‐16:Ald in blend 5 did cause a significant increase in the number of males trapped in Australia, but not in Spain or China (Fig. 5(a)). This coincides with our finding that females in Australia produced higher relative amounts Z9‐16:Ald in their pheromone blends compared to females in China and Spain, suggesting some selection for Z9‐16:Ald which may maximize attraction of conspecific males and possibly avoid interspecific matings in Australia.

Interestingly, we found significant geographic differences in male response when adding a potential sex pheromone antagonist, Z11‐16:OAc. The fact that Z11‐16:OAc dramatically inhibited attraction of *H. armigera* males in Australia and China suggests that Z11‐16:OAc is an antagonist to avoid heterospecific attraction between closely related species.^13^, ^51^, ^63^, ^70^ Specifically, *H. punctigera* co‐occurs in Australia,^71^ which also use Z11‐16:Ald as their major sex pheromone component, and has Z11‐16:OAc in their sex pheromone as well.^72^, ^73^ Similarly, *H. assulta* is sympatric with *H. armigera* in China.^74^ Even though the main sex pheromone component of *H. assulta* is Z9‐16:Ald instead of Z11‐16:Ald, the female blend also contains Z9‐16:OAc and Z11‐16:OAc.^74^, ^75^ In Spain, such communication interference does not seem to be present, as *H. armigera* males were not deterred by Z11‐16:OAc in this region.

Our findings of geographic differences in *H. armigera* male response are important for the development of region‐specific lures.^13^, ^34^ For example, using 6% of Z9‐16:Ald instead of 1.4–4% of Z9‐16:Ald in synthetic pheromone lures is probably more effective in monitoring and controlling *H. armigera* populations in Australia. Furthermore, Z11‐16:OAc has a potential application as an antagonist in pest management strategies, such as mating disruption against other closed related species in sympatric regions.

## 5 CONCLUSIONS

We found geographic variation in the sexual signals and responses of *H. armigera*, which is likely due to local environmental conditions, such photoperiod and temperature, but also due to the presence of other closely related species with which communication interference could occur. Most importantly, we found that the male response window varies in the three continents and is wider in Spain than in China and Australia. The fact that we found not only geographic variation in both the female signal and the male response indicates that sexual communication is not fixed in this species, which may have important consequences for the development of this pest in its newly invaded area of South America.

## References

[1] Luck RF , Van den Bosch R and Garcia R , Chemical insect control‐a troubled pest management strategy. Bioscience 27:606–611 (1977).

[2] Howarth FG , Environmental impacts of classical biological control. Annu Rev Entomol 36:485–509 (1991).

[3] Gentz MC , Murdoch G and King GF , Tandem use of selective insecticides and natural enemies for effective, reduced‐risk pest management. Biol Control 52:208–215 (2010).

[4] Foster SP and Harris MO , Behavioral manipulation methods for insect pest‐management. Annu Rev Entomol 42:123–146 (1997). 15012310 10.1146/annurev.ento.42.1.123

[5] Bengtsson M , Karg G , Kirsch PA , Löfqvist J , Sauer A and Witzgall P , Mating disruption of pea moth *Cydia nigricana* F. (lepidoptera: Tortricidae) by a repellent blend of sex pheromone and attraction inhibitors. J Chem Ecol 20:871–887 (1994). 24242202 10.1007/BF02059584

[6] Witzgall P , Kirsch P and Cork A , Sex pheromones and their impact on pest management. J Chem Ecol 36:80–100 (2010). 20108027 10.1007/s10886-009-9737-y

[7] Miller JR and Gut LJ , Mating disruption for the 21st century: matching technology with mechanism. Environ Entomol 44:427–453 (2015). 26313949 10.1093/ee/nvv052

[8] Rodriguez‐saona CR and Stelinski LL , Behavior‐modifying strategies in IPM: theory and practice, in Integrated Pest Management: Innovation‐Development Process, Springer, Dordrecht, pp. 263–315 (2009).

[9] Groot AT , Inglis O , Bowdridge S , Santangelo RG , Blanco C , López JD *et al*, Geographic and temporal variation in moth chemical communication. Evolution 63:1987–2003 (2009). 19473383 10.1111/j.1558-5646.2009.00702.x

[10] Unbehend M , Hänniger S , Vásquez GM , Juárez ML , Reisig D , McNeil JN *et al*, Geographic variation in sexual attraction of *Spodoptera frugiperda* corn‐ and rice‐strain males to pheromone lures. PLoS One 9:44–47 (2014). 10.1371/journal.pone.0089255 PMC3929749 24586634

[11] Ackerman JD , Geographic and seasonal variation in fragrance choices and preferences of male euglossine bees. Biotropica 21:340–347 (1989).

[12] Gleason JM and Ritchie MG , Evolution of courtship song and reproductive isolation in the *Drosophila willistoni* species complex: do sexual signals diverge the most quickly? Evolution 52:1493–1500 (1998). 28565374 10.1111/j.1558-5646.1998.tb02031.x

[13] Groot AT , Santangelo RG , Ricci E , Brownie C , Gould F and Schal C , Differential attraction of *Heliothis subflexa* males to synthetic pheromone lures in Eastern US and Western Mexico. J Chem Ecol 33:353–368 (2007). 17200888 10.1007/s10886-006-9233-6

[14] Delisle J and McNeil JN , The effect of photoperiod on the calling behaviour of virgin females of the true armyworm, *Pseudaletia unipuncta* (Haw.) (Lepidoptera: Noctuidae). J Insect Physiol 32:199–206 (1986).

[15] Noldus LPJJ and Potting RPJ , Calling behaviour of *Mamestra brassicae*: effect of age and photoperiod. Entomol Exp Appl 56:23–30 (1990).

[16] Kamimura M and Tatsuki S , Effects of photoperiodic changes on calling behavior and pheromone production in the oriental tobacco budworm moth, *Helicoverpa assulta* (Lepidoptera: Noctuidae). J Insect Physiol 40:731–734 (1994).

[17] Delisle J and McNeil JN , Calling behaviour and pheromone titre of the true armyworm *Pseudaletia unipuncta* (Haw.) (Lepidoptera: Noctuidae) under different temperature and photoperiodic conditions. J Insect Physiol 33:315–324 (1987).

[18] Webster RP and Cardé RT , Influence of relative humidity on calling behaviour of the female European corn borer moth (*Ostrinia nubilalis*). Entomol Exp Appl 32:181–185 (1982).

[19] Royer L and McNeil JN , Changes in calling behaviour and mating success in the European corn borer (*Ostrinia nubilalis*), caused by relative humidity. Entomol Exp Appl 61:131–138 (1991).

[20] Conner WE , Webster RP and Itagaki H , Calling behaviour in arctiid moths: the effects of temperature and wind speed on the rhythmic exposure of the sex attractant gland. J Insect Physiol 31:815–820 (1985).

[21] Swier SR , Rings RW and Musick GJ , Age‐related calling behavior of the black cutworm, *Agrotis ipsilon* . Ann Entomol Soc Am 70:919–924 (1977).

[22] Xavier LMS , Magalhães DM , Viana PA , Blassioli‐Moraes MC , Borges M , Barrigossi JAF , *et al*, Age influence on sexual behavior of the lesser cornstalk borer, *Elasmopalpus lignosellus* (Zeller) (Lepidoptera: Pyralidae), Neotrop Entomol 47:205–210 (2018). 28474329 10.1007/s13744-017-0527-x

[23] Ming QL , Yan YH and Wang CZ , Mechanisms of premating isolation between *Helicoverpa armigera* (Hübner) and *Helicoverpa assulta* (Guenée) (Lepidoptera: Noctuidae). J Insect Physiol 53:170–178 (2007). 17240394 10.1016/j.jinsphys.2006.11.007

[24] Landolt PJ and Phillips TW , Host plant influences on sex pheromone behavior of phytophagous insects. Annu Rev Entomol 42:371–391 (1997). 15012318 10.1146/annurev.ento.42.1.371

[25] Samudra IM , Emura K , Hoshizaki S , Ishikawa Y and Tatsuki S , Temporal differences in mating behavior between rice‐ and water‐oats‐populations of the striped stem borer, *Chilo suppressalis* (Walker)(Lepidoptera: Crambidae). Appl Entomol Zool 37:257–262 (2002).

[26] Casimero V , Nakasuji F and Fujisaki K , The influences of larval and adult food quality on the calling rate and pre‐calling period of females of the cotton bollworm, *Helicoverpa armigera* Hübner (Lepidoptera: Noctuidae). Appl Entomol Zool 36:33–40 (2001).

[27] Almeida ÂA , Lima ER and Reis R , Pupal period affects calling behavior of the wheat moth, *Pseudaletia sequax* (Lepidoptera: Noctuidae). Ethology 114:499–503 (2008).

[28] Shen LZ , Chen PZ , Xu ZH , Deng JY , Harris MK , Wanna R *et al*, Effect of larvae treated with mixed biopesticide *Bacillus thuringiensis* – abamectin on sex pheromone communication system in cotton bollworm, *Helicoverpa armigera* . PLoS One 8:e68756 (2013). 23874751 10.1371/journal.pone.0068756 PMC3706323

[29] Navarro‐Roldán MA and Gemeno C , Sublethal effects of neonicotinoid insecticide on calling behavior and pheromone production of tortricid moths. J Chem Ecol 43:881–890 (2017). 28852942 10.1007/s10886-017-0883-3

[30] McElfresh JS and Millar JG , Geographic variation in sex pheromone blend of *Hemileuca electra* from southern California. J Chem Ecol 25:2505–2525 (1999).

[31] Sadek MM , von Wowern G , Löfstedt C , Rosén WQ and Anderson P , Modulation of the temporal pattern of calling behavior of female *Spodoptera littoralis* by exposure to sex pheromone. J Insect Physiol 58:61–66 (2012). 22001286 10.1016/j.jinsphys.2011.09.016

[32] Fadamiro HY , Cossé AA and Baker TC , Fine‐scale resolution of closely spaced pheromone and antagonist filaments by flying male *Helicoverpa zea* . J Comp Physiol A 185:131–141 (1999).

[33] Miller DR , Gibson KE , Raffa KF , Seybold SJ , Teale SA and Wood DL , Geographic variation in response of pine engraver, *Ips pini*, and associated species to pheromone, lanierone. J Chem Ecol 23:2013–2031 (1997).

[34] Cruz‐Esteban S , Rojas JC , Sánchez‐Guillén D , Cruz‐López L and Malo EA , Geographic variation in pheromone component ratio and antennal responses, but not in attraction, to sex pheromones among fall armyworm populations infesting corn in Mexico. J Pest Sci 91:973–983 (2018).

[35] Wu D , Yan Y and Cui J , Sex pheromone components of *Helicoverpa armigera*: chemical analysis and field tests. Insect Sci 4:350–356 (1997).

[36] Ambrogi BG , Fonseca MG , Coracini MDA and Zarbin PHG , Calling behaviour and male response towards sex pheromone of poplar moth *Condylorrhiza vestigialis* (Lepidoptera: Crambidae). J Pest Sci 82:55–60 (2009).

[37] Rothschild GHL and Minks AK , Time of activity of male oriental fruit moths at pheromone sources in the field. Environ Entomol 3:1003–1007 (1974).

[38] Casimero V , Tsukuda R , Nakasuji F and Fujisaki K , The pre‐calling period and starting time of calling by females of three Japanese populations of the cotton bollworm, *Helicoverpa armigera* Hubner (Lepidoptera: Noctuidae). Appl Entomol Zool 34:123–127 (1999).

[39] Groot AT and Zizzari ZV , Does climate warming influence sexual chemical signaling? Anim Biol 69:83–93 (2019).

[40] Fitt GP , The ecology of *Heliothis* species in relation to agroecosystems. Annu Rev Entomol 34:17–52 (1989).

[41] Torres‐Vila LM , Rodríguez‐Molina MC , Lacasa‐Plasencia A and Bielza‐Lino P , Insecticide resistance of *Helicoverpa armigera* to endosulfan, carbamates and organophosphates: the Spanish case. Crop Prot 21:1003–1013 (2002).

[42] Czepak C and Albernaz KC , First reported occurrence of *Helicoverpa armigera* in Brazil. Pesqui Agropecu Trop 43:110–113 (2013).

[43] Ferreira Agüero MA , Gusman Sosa E and Arias OR , Identificación molecular de *Helicoverpa armígera* (Noctuidae: Heliothinae) en el departamento de Amambay, Paraguay. Invest Agrar 20:84–90 (2018).

[44] Tay WT , Soria MF , Walsh T , Thomazoni D , Silvie P , Behere GT , *et al*, A brave new world for an old world pest: *Helicoverpa armigera* (Lepidoptera: Noctuidae) in Brazil, PLoS One 8:e80134 (2013). 24260345 10.1371/journal.pone.0080134 PMC3832445

[45] Kriticos DJ , Ota N , Hutchison WD , Beddow J , Walsh T , Tay WT *et al*, The potential distribution of invading *Helicoverpa armigera* in North America: is it just a matter of time? PLoS One 10:1–24 (2015). 10.1371/journal.pone.0119618 PMC4364701 25786260

[46] Cunningham JP and Zalucki MP , Understanding heliothine (Lepidoptera: Heliothinae) pests: what is a host plant? J Econ Entomol 107:881–896 (2014). 25026644 10.1603/ec14036

[47] Zalucki MP and Furlong MJ , Forecasting *Helicoverpa* populations in Australia: a comparison of regression based models and a bioclimatic based modelling approach. Insect Sci 12:45–56 (2005).

[48] Jallow MFA and Zalucki MP , Within‐and between‐population variation in host‐plant preference and specificity in Australian *Helicoverpa armigera* (Hubner)(Lepidoptera: Noctuidae). Aust J Zool 44:503–519 (1996).

[49] Jallow MFA , Paul Cunningham J and Zalucki MP , Intra‐specific variation for host plant use in *Helicoverpa armigera* (Hübner) (Lepidoptera: Noctuidae): implications for management. Crop Prot 23:955–964 (2004).

[50] Madhu TN , Shah VK , Prabhulinga T , Chakravarthy AK and Ashok Kumar CT , Optimization of pheromone trap densities and impact of insecticides on pheromone catches for mass trapping *Helicoverpa armigera* (Hubner) (Lepidoptera: Noctuidae) in chickpea. J Entomol Zool Stud 7:78–84 (2019).

[51] Xu M , Guo H , Hou C , Wu H , Huang LQ and Wang CZ , Olfactory perception and behavioral effects of sex pheromone gland components in *Helicoverpa armigera* and *Helicoverpa assulta* . Sci Rep 6:1–14 (2016). 26975244 10.1038/srep22998 PMC4792173

[52] McNeil JN , Behavioral ecology of pheromone‐mediated communication in moths and its importance in the use of pheromone traps. Annu Rev Entomol 36:407–430 (1991).

[53] Zhou X , Coll M and Applebaum SW , Effect of temperature and photoperiod on juvenile hormone biosynthesis and sexual maturation in the cotton bollworm, *Helicoverpa armigera*: implications for life history traits. Insect Biochem Mol Biol 30:863–868 (2000). 10876131 10.1016/s0965-1748(00)00059-x

[54] Zhao XC , Wu KM and Guo YY , Comparisons of calling behaviour of different geographical populations of *Helicoverpa armigera* . J Appl Entomol 131:684–689 (2007).

[55] Ono T , Charlton RE and Cardé RT , Variability in pheromone composition and periodicity of pheromone titer in potato tuberworm moth, *Phthorimaea operculella* (Lepidoptera: Gelechiidae). J Chem Ecol 16:531–542 (1990). 24263509 10.1007/BF01021784

[56] Delisle J and Royer L , Changes in pheromone titer of oblique‐banded leafroller, *Choristoneura rosaceana*, virgin females as a function of time of day, age, and temperature. J Chem Ecol 20:45–69 (1994). 24241698 10.1007/BF02065990

[57] Xiang YY , Yang MF and Li ZZ , Calling behavior and rhythms of sex pheromone production in the black cutworm moth in China. J Insect Behav 23:35–44 (2010).

[58] Symonds MRE , Johnson TL and Elgar MA , Pheromone production, male abundance, body size, and the evolution of elaborate antennae in moths. Ecol Evol 2:227–246 (2012). 22408739 10.1002/ece3.81 PMC3297191

[59] Barthel A , Staudacher H , Schmaltz A , Heckel DG and Groot AT , Sex‐specific consequences of an induced immune response on reproduction in a moth. BMC Evol Biol 15:1–12 (2015). 26672978 10.1186/s12862-015-0562-3 PMC4681174

[60] Firempong S and Zalucki MP , Host plant preferences of populations of *Helicoverpa armigera* (Hubner)(Lepidoptera, Noctuidae) from different geographic locations. Aust J Zool 37:665–673 (1989).

[61] Liu Z , Li D , Gong P and Wu K , Life table studies of the cotton bollworm, *Helicoverpa armigera* (Hübner) (Lepidoptera: Noctuidae), on different host plants. Environ Entomol 33:1570–1576 (2004).

[62] Konyukhov VP , Kovalev BG and Sammar Zade NR , Isolation and identification of the components of the sex pheromone of the corn earworm *Heliothis armigera* Hb. Sov J Bioorg Chem 9:782–787 (1983).

[63] Löfstedt C , Herrebout WM and Menken SB , Sex pheromones and their potential role in the evolution of reproductive isolation in small ermine moths (Ypnomeutidae). Chem 2:20–28 (1991).

[64] Groot AT , Nojima S , Heath JJ , Ammagarahalli B , van Wijk M , Claβen A , *et al*, Alcohol contributes to attraction of *Heliothis* (= *Chloridea*) *virescens* males to females, J Chem Ecol 44:621–630 (2018). 30039209 10.1007/s10886-018-0995-4

[65] Dunkelblum E , Gothilf S and Kehat M , Identification of the sex pheromone of the cotton bollworm, *Heliothis armigera*, in Israel. Phytoparasitica 8:209–211 (1980).

[66] Kehat M , Gothilf S , Dunkelblum E and Greenberg S , Field evaluation of female sex pheromone components of the cotton bollworm, *Heliothis armigera* . Entomol Exp Appl 27:188–193 (1980).

[67] Kehat M and Dunkelblum E , Behavioral responses of male *Heliothis armigera* (Lepidoptera: Noctuidae) moths in a flight tunnel to combinations of components identified from female sex pheromone glands. J Insect Behav 3:75–83 (1990).

[68] Piccardi P , Capizzi A , Cassani G , Spinelli P , Arsura E and Massardo P , A sex pheromone component of the old world bollworm *Heliothis armigera* . J Insect Physiol 23:1443–1445 (1977).

[69] Zhang JP , Salcedo C , Fang YL , Zhang RJ and Zhang ZN , An overlooked component: (Z)‐9‐tetradecenal as a sex pheromone in *Helicoverpa armigera* . J Insect Physiol 58:1209–1216 (2012). 22732233 10.1016/j.jinsphys.2012.05.018

[70] Cardé RT , Cardé AM , Hill AS and Roelofs WL , Sex pheromone specificity as a reproductive isolating mechanism among the sibling species *Archips argyrospilus* and *A. mortuanus* and other sympatric tortricine moths (Lepidoptera: Tortricidae). J Chem Ecol 3:71–84 (1977).

[71] Zalucki MP , Daglish G , Firempong S and Twine P , The biology and ecology of *Heliothis armigera* (Hubner) and *Heliothis punctigera* Wallengren (Lepidoptera, Noctuidae) in Australia – what do we know. Aust J Zool 34:779–814 (1986).

[72] Rothschild GHL , Attractants for *Heliothis armigera* and *H. punctigera* . J Aust Entomol Soc 17:389–390 (1978).

[73] Rothschild GHL , Nesbitt BF , Beevor PS , Cork A , Hall DR and Vickers RA , Studies of the female sex pheromone of the native budworm, *Heliothis punctigera* . Entomol Exp Appl 31:395–401 (1982).

[74] Wang HL , Zhao CH and Wang CZ , Comparative study of sex pheromone composition and biosynthesis in *Helicoverpa armigera*, *H. assulta* and their hybrid. Insect Biochem Mol Biol 35:575–583 (2005). 15857763 10.1016/j.ibmb.2005.01.018

[75] Cork A , Boo KS , Dunkelblum E , Hall DR , Jee‐Rajunga K , Kehat M , *et al*, Female sex pheromone of oriental tobacco budworm, *Helicoverpa assulta* (Guenee)(Lepidoptera: Noctuidae): identification and field testing, J Chem Ecol 18:403–418 (1992). 24254945 10.1007/BF00994240

